# A High-Performance Deep Neural Network Model for BI-RADS Classification of Screening Mammography

**DOI:** 10.3390/s22031160

**Published:** 2022-02-03

**Authors:** Kuen-Jang Tsai, Mei-Chun Chou, Hao-Ming Li, Shin-Tso Liu, Jung-Hsiu Hsu, Wei-Cheng Yeh, Chao-Ming Hung, Cheng-Yu Yeh, Shaw-Hwa Hwang

**Affiliations:** 1 Department of General Surgey, E-Da Cancer Hospital, Yanchao Dist., Kaohsiung 82445, Taiwan; tsai560612@gmail.com (K.-J.T.); ed100647@edah.org.tw (C.-M.H.); 2 College of Medicine, I-Shou University, Yanchao Dist., Kaohsiung 82445, Taiwan; 3 Department of Radiology, E-Da Hospital, Yanchao Dist., Kaohsiung 82445, Taiwan; meichun@gmail.com (M.-C.C.); lhm0323@gmail.com (H.-M.L.); ed106088@edah.org.tw (S.-T.L.); wenbear0330@gmail.com (J.-H.H.); 4 Department of Radiology, E-Da Cancer Hospital, Yanchao Dist., Kaohsiung 82445, Taiwan; ywcywcywcywc@gmail.com; 5 Department of Electrical Engineering, National Chin-Yi University of Technology, Taichung 41170, Taiwan; 6 Department of Electrical and Computer Engineering, National Yang Ming Chiao Tung University, Hsinchu 30010, Taiwan; hsf@nctu.edu.tw

**Keywords:** screening mammography, breast imaging reporting and data system (BI-RADS), image classification, deep neural network (DNN), deep learning

## Abstract

Globally, the incidence rate for breast cancer ranks first. Treatment for early-stage breast cancer is highly cost effective. Five-year survival rate for stage 0–2 breast cancer exceeds 90%. Screening mammography has been acknowledged as the most reliable way to diagnose breast cancer at an early stage. Taiwan government has been urging women without any symptoms, aged between 45 and 69, to have a screening mammogram bi-yearly. This brings about a large workload for radiologists. In light of this, this paper presents a deep neural network (DNN)-based model as an efficient and reliable tool to assist radiologists with mammographic interpretation. For the first time in the literature, mammograms are completely classified into BI-RADS categories 0, 1, 2, 3, 4A, 4B, 4C and 5. The proposed model was trained using block-based images segmented from a mammogram dataset of our own. A block-based image was applied to the model as an input, and a BI-RADS category was predicted as an output. At the end of this paper, the outperformance of this work is demonstrated by an overall accuracy of 94.22%, an average sensitivity of 95.31%, an average specificity of 99.15% and an area under curve (AUC) of 0.9723. When applied to breast cancer screening for Asian women who are more likely to have dense breasts, this model is expected to give a higher accuracy than others in the literature, since it was trained using mammograms taken from Taiwanese women.

## 1. Introduction

Globally, the incidence rate for breast cancer ranks first [1]. A recent report [2] indicates that more than 10,000 Taiwanese women are diagnosed as having breast cancer, and more than 2000 died of breast cancer in 2018. As a matter of fact, treatments for early-stage breast cancer are effective. The 5-year survival rate for stage 0–2 breast cancer exceeds 90%, while it falls below 25% for stage 4 [3]. Screening mammography has been acknowledged as the most reliable way to detect breast cancer at an early stage, particularly in detecting grouped micro-calcification lesions. For years, the Taiwanese government has been urging women without any symptoms, aged between 45 and 69, to have a screening mammogram on a biennial basis. A great number of mammograms are collected in a large-scale mammography screening program and need to be interpreted by well-qualified but overloaded radiologists. Hence, there is definitely an unmet need to develop AI models to assist radiologists with mammographic interpretation, and AI model development requires interdisciplinary research that integrates medical science and engineering.

Routine screening mammography consists of the cranio-caudal (CC) view and the mediolateral-oblique (MLO) view of each breast of a woman, that is, the LCC, RCC, LMLO and RMLO views in total. Developed by the American College of Radiology (ACR), the Breast Imaging Reporting and Data System (BI-RADS) [4] lexicon is used to standardize the reporting of mammographic findings, assessment categories and follow-up management, and communication between radiologists and referring physicians can be facilitated accordingly.

As referenced previously, it takes radiologists an enormous amount of effort to interpret screening mammograms. Therefore, a great amount of computer-aided detection (CAD) systems have been developed to improve the efficiency of mammogram interpretation. Recently, deep learning models have been used to measure the likelihood of cancer from a mammogram [5,6,7,8,9,10,11,12,13,14,15,16,17,18,19,20,21,22], some of which were designed to detect and classify micro-calcifications or calcified lesions [8,9,10], mass lesions [11,12,13,14] and even all the contained lesions [15,16,17,18,19,20,21,22]. Breast lesions found in mammograms are mainly classified into normal, benign and malignant [15,16,17,18,19,20] and are further classified as normal, benign calcification, benign mass, malignant calcification and malignant mass [21,22].

BI-RADS classification is frequently used in breast cancer screening. Therefore, there is definitely a necessity to develop AI models for efficient and reliable BI-RADS classification. However, little has been reported on this issue in the literature so far, mainly due to an inadequate number of open-access mammogram datasets. For example, breast masses were classified incompletely into BI-RADS categories 2–5 using a computer-aided diagnosis system [13] where merely 300 mammograms were employed as training data, and another 200 mammograms were employed as testing data.

Accordingly, this paper presents a deep learning model to address the BI-RADS-classification issue. Breast lesions were classified into categories 0, 1, 2, 3, 4A, 4B, 4C and 5, but excluding category 6, which indicates a known biopsy-proven malignancy. For the first time in the literature, breast lesions can be completely classified using a deep learning model that was well trained by a mammogram dataset of our own. For the purpose of model training, all the lesions contained were labeled and classified by six well-qualified radiologists, as will be detailed below.

It is worth mentioning that this work can provide at least three benefits for medical industries. First, this developed tool can assist radiologists with mammographic interpretation in clinical works and can improve the efficiency of mammogram interpretation as well. Second, the workload of radiologists can be significantly eased, particularly when interpreting mammograms in a large-scale breast cancer screening program. Third, the tool can assist general physicians to interpret mammograms due to there being a shortage of radiologists or breast surgeons in most remote areas.

This paper is outlined as follows. Section 2 describes a labeled and annotated mammogram dataset for training purposes. Section 3 presents a deep neural network (DNN)-based model for BI-RADS classification. Experimental results and discussions are given in Section 4. Finally, Section 5 concludes this study.

## 2. Materials and Lesion Annotation

Firstly, Table 1 gives the complete BI-RADS categories, the respective description and assessment of mammography [23]. As can be found therein, category 4 is further sub-categorized into categories 4A, 4B and 4C to indicate the different levels of malignancy suspicion.

The digital mammogram dataset employed in this work is provided by the E-Da hospital, Taiwan. The dataset is composed of up to 5733 mammograms of 1490 patients, including 1434 LCC, 1436 RCC, 1433 LMLO and 1430 RMLO views, within the time frame of 2004 and 2010. This study was approved by a local institutional review board (EMRP-108-142), and informed consent was waived. This is simply because there is no personal identifiable data in the dataset, since all the personal data were deleted.

**Figure 1:**
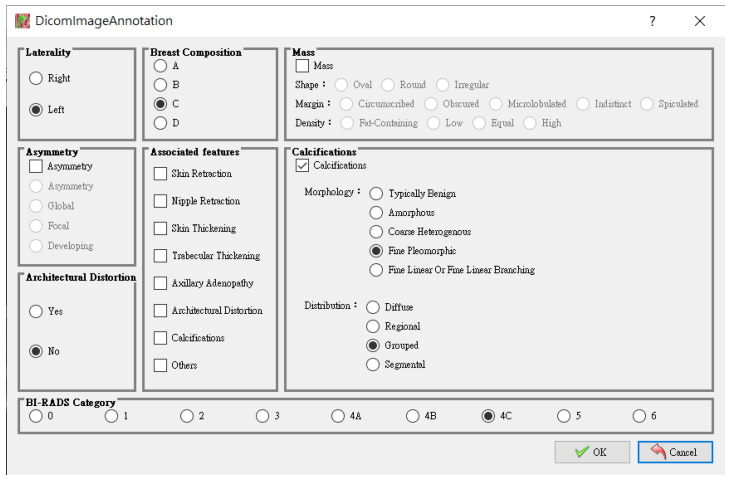
An interface for breast lesion annotation.

**Figure 2:**
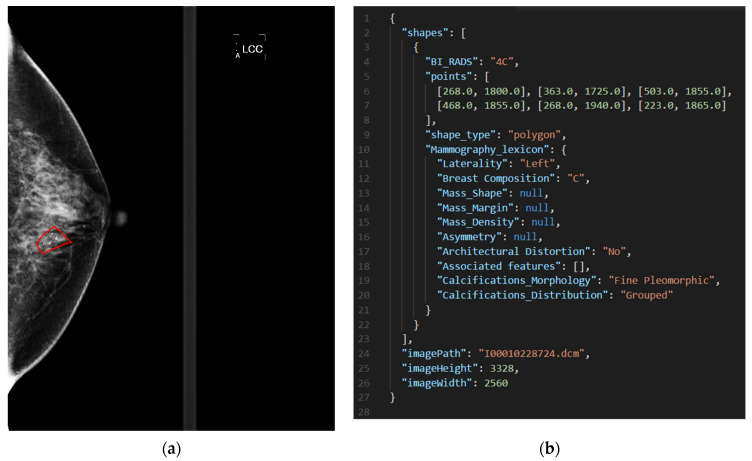
(**a**) A BI-RADS category 4C mammogram with a labeled lesion and (**b**) a JSON file that saved the annotation in (**a**).

To facilitate data preprocessing, an easy-to-use tool was exclusively developed for users to label the lesion in each mammogram. Once the image labeling was completed, an interface, as illustrated in Figure 1, appeared to give users detailed annotation. In this work, all the lesions in the mammograms were labeled by a total of six qualified radiologists of the E-Da hospital. The annotation was saved as a JSON file. For illustrative purposes, Figure 2 gives a BI-RADS category 4C mammogram with a labeled lesion and shows a JSON file that saved the annotation in Figure 1.

Table 2 gives the statistics on the number of lesion annotations. As can be found therein, there is no annotation in BI-RADS category 1, simply because category 1 means that the breast tissue looked healthy, and there was no need to annotate accordingly. Additionally, there is a maximum of 8 annotations in a mammogram and a total of 4557 annotations for all the mammograms in this work.

## 3. Methodology and Model

**Figure 3:**
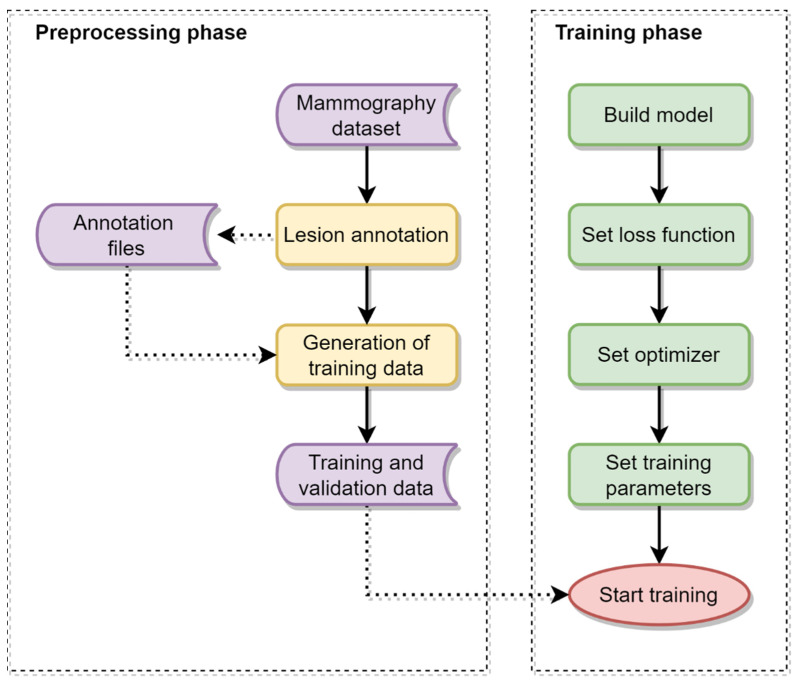
Flowcharts of the preprocessing and training phase in this work.

This paper presents a DNN-based model to classify mammograms into categories 0, 1, 2, 3, 4A, 4B, 4C and 5, but excluding category 6, since category 6 is used to represent a female diagnosed with breast cancer. As illustrated in Figure 3, the model was trained using block-based images segmented from the dataset. A block-based image was applied to the model as an input, and a category was assigned as an output. In this manner, the feature maps of the block-based images were correlated with the BI-RADS categories.

The DNN-based model has the following advantages. It was well trained using a multitude of block images, and it is the first time that mammograms were classified into eight BI-RADS categories for the sake of completeness in the literature. Finally, breast lesions can be reliably located and efficiently classified to allow the radiologists to speed up mammogram interpretation. The training data and the flowchart of the presented model are described as follows.

### 3.1. Block Images as Training Data

**Figure 4:**
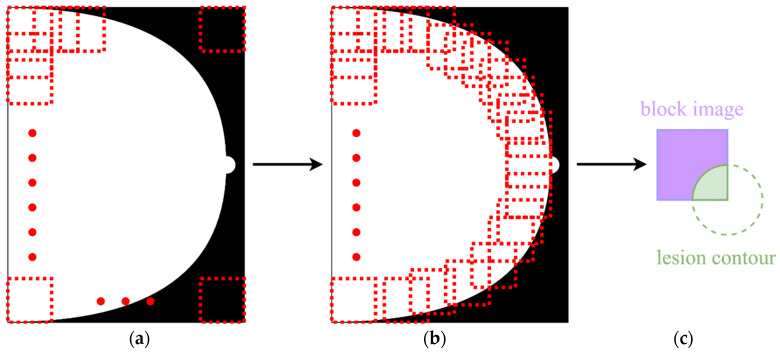
(**a**) Overlapping block images, (**b**) those of (**a**) selected as training data, and (**c**) a BI-RADS category assigned to each block image in (**b**).

As referenced previously, the presented model was trained using a multitude of block-based images of size 224 × 224 pixels in this work. Figure 4 illustrates block images and a lesion contained in a block image. As illustrated in Figure 4a,b, the white portions represent the same view of a breast, and a mammogram is segmented into overlapping block images from right to left and then top to bottom, with a stride of 36 pixels. Furthermore, a block image where a contained breast occupies no less than 90% of the block area is chosen as a piece of training data.

As illustrated in Figure 4c, part of a lesion is contained in the block image. Next, a BI-RADS category is assigned to the block image according to the ratio of the areas of the contained lesion to the area of the block, which can be categorized as follows. In Case 1, a block image does not contain a lesion and is assigned as BI-RADS category 1 accordingly. Otherwise, two quantities, *ratio*_B_ and *ratio*_L_ are, respectively defined in Case 2 as

(1) $$ratio_{B}=\frac{Area_{B}\cap^{\;}Area_{L}}{Area_{B}}$$

(2) $$ratio_{L}=\frac{Area_{B}\cap^{\;}Area_{L}}{Area_{L}}$$

where *Area*_B_ and *Area*_L_ represent the areas of the block image and the lesion, respectively. Subsequently, if the condition

Condition: (*ratio*_B_ ≥ *thr*_B_) or (*ratio*_L_ ≥ *thr*_L_) (3)

where *thr*_B_ = *thr*_L_ = 0.5 are two user-specified thresholds, is true, the block image is then classified as the category of the contained lesion. In Case 3, where there are multiple findings in a block image, check whether the condition in Expression (3) is satisfied. If satisfied, the block image is assigned the highest category in the following hierarchy, from highest to lowest: 5, 4C, 4B, 4A, 0, 3, 2. Otherwise, the block image is assigned as BI-RADS category 1. All the block images were divided into two parts, as the training and test data, respectively, and Table 3 gives the numbers of these data for each BI-RADS category.

### 3.2. Model Architecture

**Figure 5:**
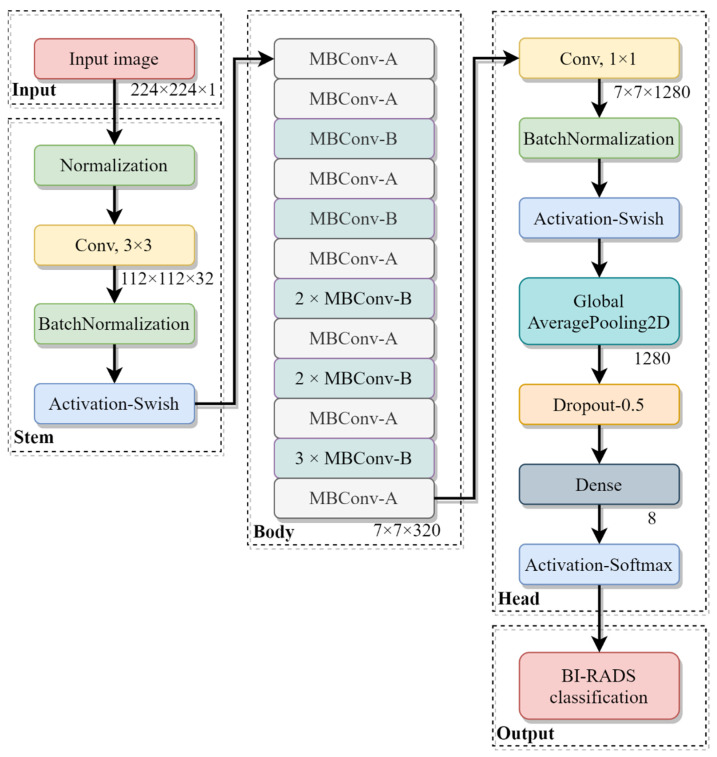
Flowchart of the presented BI-RADS classification model.

The model was built based on one of the state-of-the-art models, EfficientNet [24]. As illustrated in Figure 5, the model, made up of a stem, a body, a head and an output mode, takes a mammogram of size 224 × 224 pixels as an input, that is, an input image shape of 224 × 224 × 1. In the Stem module, the input image is firstly normalized to lie between 0 and 1, and then feature maps are extracted using a 3 × 3 convolution layer. Subsequently, high-level feature maps are extracted in the Body module, consisting of 16 mobile inverted bottleneck convolution (MBConv) blocks [25]. Finally, the feature maps are classified in the Head and Output modules.

A Swish activation function [26], expressed as

(4) $$Swish\left(x,\;\beta \right)=x\cdot sigmoid\left(\beta x\right)=\frac{x}{1+e^{-\beta x}}$$

is used in the Activation-Swish block. As compared with ReLU, the performance of a neural network can be improved in most cases using a Swish activation function. Table 4 summarizes all the modules contained in Figure 5.

**Figure 6:**
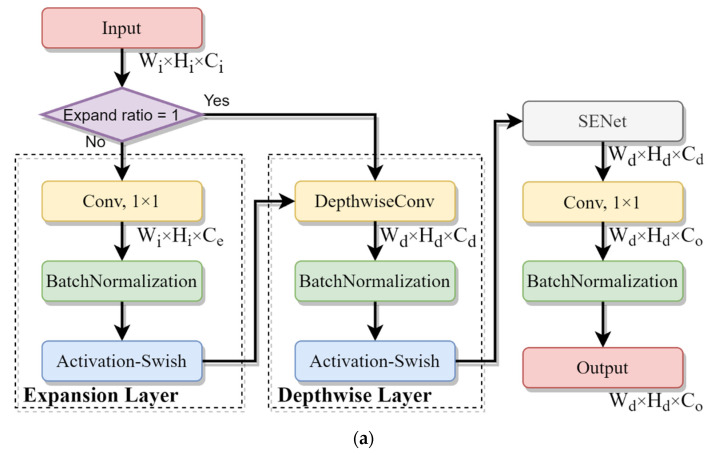

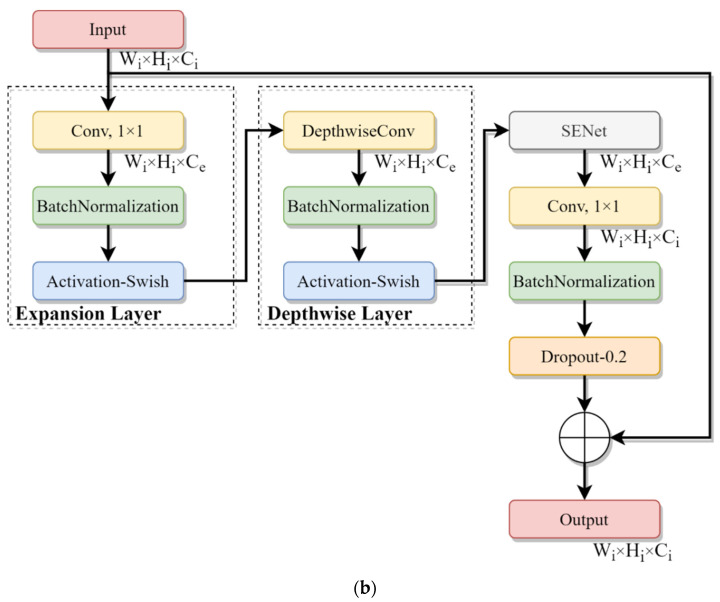
Flowcharts of (**a**) the MBConv-A block and (**b**) the MBConv-B block.

Figure 6 gives detailed flowcharts of the MBConv-A and B blocks in Figure 5. An MBConv block is mainly composed of an expansion layer, a depthwise layer and a squeeze-and-excitation network (SENet) [27] where *C*_e_ = *C*_i_ × *R*_e_, and *R*_e_ represents the expansion ratio, as tabulated in Table 4. Accordingly, *C*_d_ = *C*_i_ if *R*_e_ = 1, and *C*_d_ = *C*_e_ otherwise. Additionally, Table 4 gives the kernel size and the stride for each DepthwiseConv. For stride = 1, the output shape is equal to the input shape of a feature map, that is, (*W*_d_, *H*_d_) = (*W*_i_, *H*_i_). For stride = 2, the output shape is half of the input shape. The values of the parameters *W*_d_, *H*_d_ and *C*_o_ can be referenced in Table 4.

**Figure 7:**
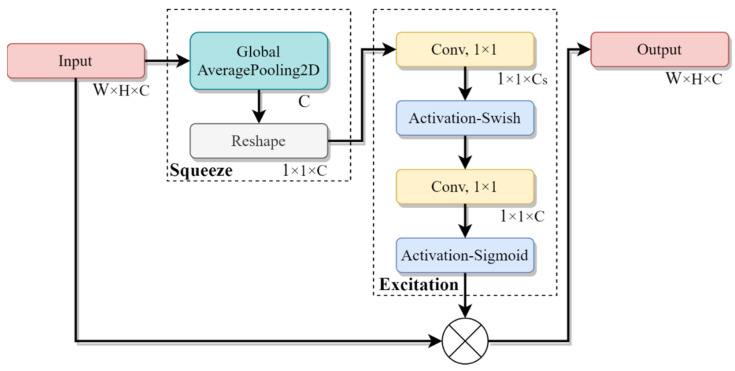
Flowchart of the SENet module.

The SENet module is detailed in Figure 7. A feature map is downsized from W × H × C to 1 × 1 × C in the squeeze module. To take arbitrary-sized feature map as an input, two fully connected layers are replaced with two convolutional layers with a kernel size of 1 × 1 in the excitation module, and *C*_s_ = *C*_i_ × *R*_s_ where *C*_i_ represents the one in the MBConv block, and *R*_s_ represents a user-specified ratio that is set to 0.25. Each channel of the input is weighted non-uniformly by multiplying the input and the output of the excitation module, so as to reflect the significance of each channel feature.

Finally, a categorical cross-entropy loss function was used to train the model with a batch size of 128 and an epoch of 350, and a Ranger optimizer [28] was also used to improve the training performance. Table 5 lists the development environment of this work.

## 4. Experimental Results

A confusion matrix for an eight-class classification system and four performance metrics for each class, including the sensitivity, specificity, precision and F1-score, were evaluated to quantify the model performance. Then, the mean value of each performance metric and the overall accuracy were found.

**Figure 8:**
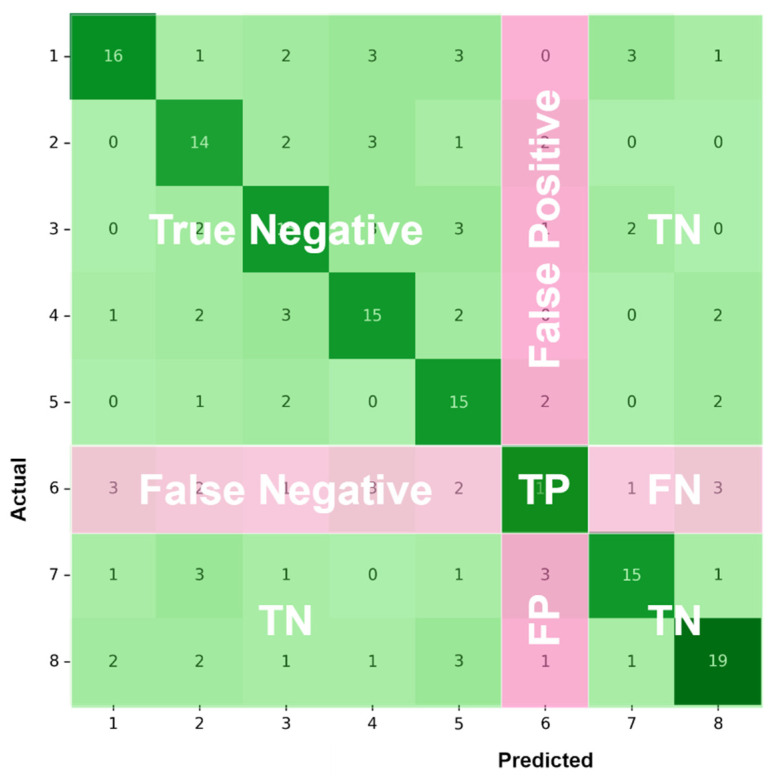
An 8 × 8 confusion matrix for illustrative purposes.

In Figure 8, an 8 × 8 confusion matrix is used to illustrate how all the performance metrics were evaluated in the case of type 6 (BI-RADS category 4B). True positive (TP) and false positive (FP) are used to represent a lesion that is accurately classified or misclassified as category 4B, respectively. Likewise, true negative (TN) and false negative (FN) are used to represent a lesion that is accurately classified or misidentified as a category, other than category 4B, respectively.

Accordingly, all the performance metrics are given, respectively, by

*Sensitivity*_k_ = *TPR*_k_ = *TP*_k_/(*TP*_k_ + *FN*_k_) (5)

*Specificity*_k_ = *TNR*_k_ = *TN*_k_/(*TN*_k_ + *FP*_k_) (6)

*Precision*_k_ = *PPV*_k_ = *TP*_k_/(*TP*_k_ + *FP*_k_) (7)

*F1-score*_k_ = 2 × (*Precision*_k_ × *Sensitivity*_k_)/(*Precision*_k_ + *Sensitivity*_k_) (8)

where 1 ≤ *k* ≤ *CNum* = 8 and is used to represent that a lesion is classified as category *l* = the *k*th element of the hierarchy: 0, 1, 2, 3, 4A, 4B, 4C, 5, e.g., category 2 for *k* = 3. The sensitivity, specificity and precision are also referred to as the true positive rate (TPR), true negative rate (TNR) and positive predictive value (PPV), respectively. The mean values of the performance metrics in Equations (5)–(8) and the overall accuracy are respectively given by

(9) $$mean\left(x\right)=\frac{1}{CNum}\;\overset{CNum}{\underset{k=1}{\sum }}x_{k},\;x\in \left\{Sensitivity,\;Specificity,\;Precision,\;F1-score\right\}$$

(10) $$Accuracy=\frac{\sum_{k=1}^{CNum}TP_{k}}{TNum}=\frac{\sum_{k=1}^{CNum}TP_{k}}{\sum_{k=1}^{CNum}\left(TP_{k}+FN_{k}\right)}$$

where *TNum* represents the number of the test data.

**Figure 9:**
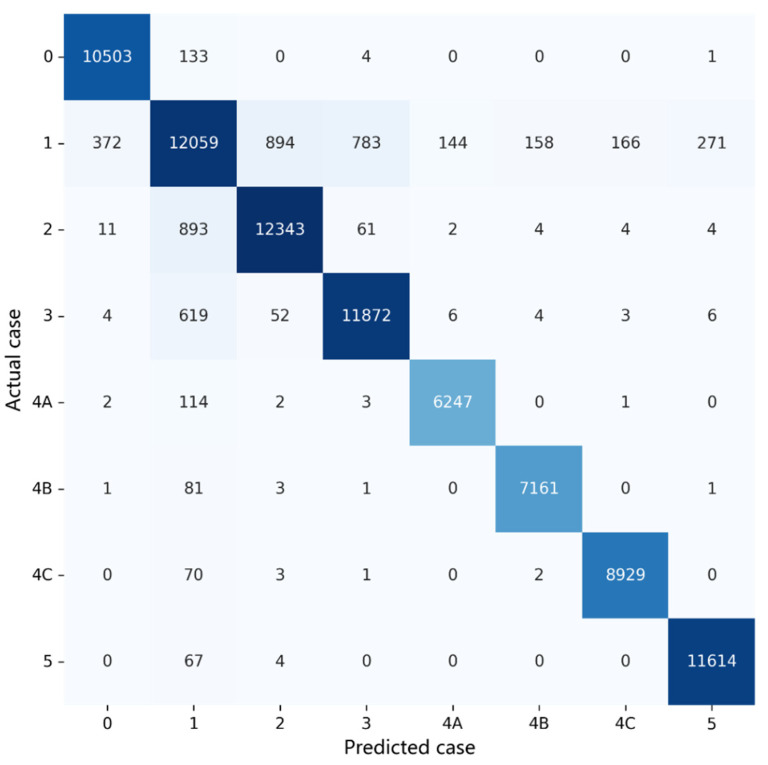
A confusion matrix for performance analysis.

**Figure 10:**
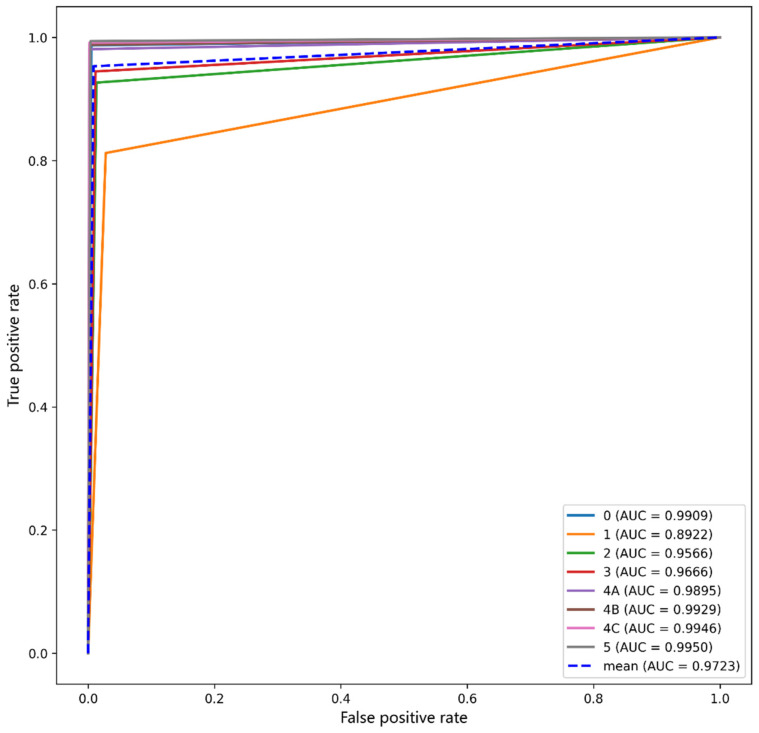
ROC curves of the performance metrics.

Performance testing was conducted using the 85,683 pieces of test data, as tabulated in Table 3, and led to the confusion matrix in Figure 9 and the performance metrics in Table 6. Subsequently, a receiver operating characteristic (ROC) curve was plotted for each BI-RADS category in Figure 10, and the corresponding area under curve (AUC) value was shown therein. The outperformance of this work was clearly indicated by an average sensitivity of 95.31%, an average specificity of 99.15%, an average precision of 94.93%, an average F1-score of 95.11%, an average AUC of 97.23% and an overall accuracy of up to 94.22%.

In each case of BI-RADS category 0, 4A, 4B, 4C and 5 lesions, the sensitivity, specificity and precision exceeded 98%, 99% and 96%, respectively. This validates that such lesions can be well classified using this work, and early-stage breast cancer can be diagnosed more accurately.

In the cases of BI-RADS category 2 and 3 lesions, all the performance metrics lay above 92%, which was slightly below those in the above-referred five cases. The worst performance occurred in the case of BI-RADS category 1 lesions, and the sensitivity and precision hit 81.22% and 85.91%, respectively, for the following reason. All the lesion-free block images were classified as BI-RADS category 1, leading to non-distinctive features that were difficult to diagnose.

A deeper investigation revealed that the sensitivity in the BI-RADS category 1 case was actually a function of the thresholds *thr*_B_ and *thr*_L_ in Equation (3). This is because a block image, classified as BI-RADS category 1, in fact contained a small portion of a lesion in some cases, leading to a negative effect on the training of the presented model. Additionally, each performance metric is also a function of *thr*_B_ and *thr*_L_.

**Figure 11:**
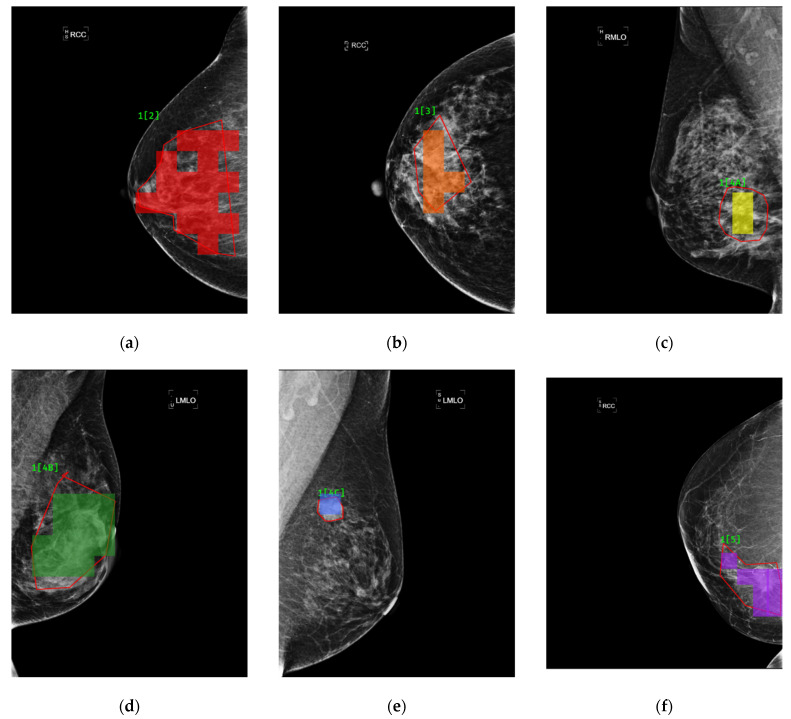
Comparisons between findings labeled by radiologists (framed in red) and highlighted in color in the cases of BI-RADS category 2, 3, 4A, 4B, 4C and 5 lesions in (**a**–**f**), respectively.

The outperformance of this model was indicated by an overall accuracy of 94.22%, an average sensitivity of 95.31% and an average specificity of 99.15%. As can be found in Figure 11, there is a good agreement between the red framed ground truth and the blocks, highlighted in color, in each of the mammograms in Figure 11a–f, where findings were classified as BI-RADS categories 2–5, respectively.

Finally, Table 7 lists the task and performance comparisons between the presented study and previous studies on breast cancer detection in order to reveal the contribution of this work. The Ave_Sen, Ave_Spe and Acc represent the average sensitivity, average specificity and accuracy, respectively.

## 5. Conclusions

This paper presented a DNN-based model to efficiently and reliably locate and classify breast lesions from mammograms. Block-based images, segmented from collected mammograms, were used to adequately train the model, by which the workload of radiologists can be significantly eased, particularly when interpreting mammograms in a large-scale breast cancer screening program. For the first time in the literature, breast lesions can be completely classified into BI-RADS categories 0, 1, 2, 3, 4A, 4B, 4C and 5. The outperformance of this model was indicated by an overall accuracy of 94.22%, an average sensitivity of 95.31%, an average specificity of 99.15% and an average AUC of 0.9723. When applied to breast cancer screening for Asian women, who are more likely to have dense breasts, this model is expected to give a higher accuracy than others in the literature, since it was trained using mammograms taken from Taiwanese women.

It is worth mentioning that this work can provide three benefits for healthcare industries. First, the developed tool can help radiologists with mammographic interpretation in clinical works and can improve the efficiency of mammogram interpretation as well. Second, the workload of radiologists can be reduced remarkably. Third, the tool can assist general physicians with interpreting mammograms due to a shortage of radiologists or breast surgeons in most remote areas.

As the next step, our team aims to upsize the collected dataset so as to better train the model and advance the generalization ability as well. In the meantime, we are making continuous efforts to improve the model performance, particularly in the worst BI-RADS category 1 case. Finally, we will test the generalization ability of this model as an inter-hospital project.

## Figures and Tables

**Table 1:** Description and assessment of BI-RADS categories for mammograms.

BI-RADS	Definition	Management	Likelihood of Cancer
0	Incomplete, need additional imaging evaluation	Recall for additional imaging and/or awaiting prior examinations	–
1	Negative (normal)	Routine screening	0%
2	Benign	Routine screening	0%
3	Probably benign	Short-interval follow-up or continued	>0% to ≤2%
4A	Low suspicion of malignancy	Tissue diagnosis	>2% to ≤10%
4B	Moderate suspicion of malignancy	Tissue diagnosis	>10% to ≤50%
4C	High suspicion of malignancy	Tissue diagnosis	>50% to <95%
5	Highly suggestive of malignancy	Tissue diagnosis	≥95%
6	Known biopsy-proven malignancy	Surgical excision when clinically appropriate	100%

**Table 2:** Number of lesion annotations in each BI-RADS category.

BI-RADS	Number of Annotations
0	520
1	0
2	2125
3	847
4A	367
4B	277
4C	217
5	204
Overall	4557

**Table 3:** Numbers of training and test data.

BI-RADS	Number of Training Data	Number of Test Data
0	42,565	10,641
1	51,964	14,847
2	48,294	13,322
3	47,470	12,566
4A	25,475	6369
4B	28,993	7248
4C	36,021	9005
5	46,741	11,685
Sum	327,523	85,683

**Table 4:** Summary of each module in the presented model.

Module	Kernel Size	Stride	Expansion Ratio	Parameters	Output Shape
Stem	3 × 3	2	−	416	(None, 112, 112, 32)
MBConv-A	3 × 3	1	1	1544	(None, 112, 112, 16)
MBConv-A	3 × 3	2	6	6436	(None, 56, 56, 24)
MBConv-B	3 × 3	1	6	11,334	(None, 56, 56, 24)
MBConv-A	5 × 5	2	6	16,006	(None, 28, 28, 40)
MBConv-B	5 × 5	1	6	32,330	(None, 28, 28, 40)
MBConv-A	3 × 3	2	6	38,250	(None, 14, 14, 80)
2 × MBConv-B	3 × 3	1	6	209,960	(None, 14, 14, 80)
MBConv-A	5 × 5	1	6	128,148	(None, 14, 14, 112)
2 × MBConv-B	5 × 5	1	6	422,968	(None, 14, 14, 112)
MBConv-A	5 × 5	2	6	265,564	(None, 7, 7, 192)
3 × MBConv-B	5 × 5	1	6	1,778,832	(None, 7, 7, 192)
MBConv-A	3 × 3	1	6	722,480	(None, 7, 7, 320)
Head	1 × 1	1	−	424,968	(None, 8)

**Table 5:** Development environment.

**Programing Language**	**Python**
Library	TensorFlow, Keras, numpy, OpenCV, etc.
Hardware	PC (Windows 10 64-bit, Intel i7-10700 2.9 GHz CPU, 128 GB RAM), graphics card (GeForce RTX 3090)

**Table 6:** Performance metrics of the proposed model.

BI-RADS	Sensitivity (%)	Specificity (%)	Precision (%)	F1-Score (%)
0	98.7031	99.4803	96.4197	97.5481
1	81.2218	97.2090	85.9148	83.5024
2	92.6513	98.6761	92.7975	92.7243
3	94.4772	98.8334	93.2967	93.8832
4A	98.0845	99.8084	97.6246	97.8540
4B	98.7997	99.7858	97.7077	98.2507
4C	99.1560	99.7731	98.0885	98.6194
5	99.3924	99.6176	97.6212	98.4989
Mean	95.3107	99.1480	94.9339	95.1101
Accuracy (%)	94.2171			

**Table 7:** Task and performance comparisons between the presented study and previous studies on breast cancer detection.

Reference (Year)	Task	Dataset Used	Ave_Sen (%)	Ave_Spe (%)	Acc (%)	AUC
This study (2022)	Classification of BI-RADS 0, 1, 2, 3, 4A, 4B, 4C, 5 (8 categories)	Private (1490 cases, 5733 images)	95.31	99.15	94.22	0.972
[8] (2021)	Malignancy prediction of BI-RADS 4 micro-calcifications (2 classes)	Private (384 cases, 824 images)	85.3	91.9	-	0.910
[11] (2021)	Mass malignancy classification (2 classes)	DDSM (2578 cases, 10,312 images)	89.8 @ 2 FPPI ^1^	-	-	-
		Private (2807 cases, 11,228 images)	96.2 @ 2 FPPI	-	-	-
[13] (2020)	BI-RADS 2-5 classification for breast masses (4 categories)	DDSM (500 images)	84.5	94.25	84.5	-
[22] (2019)	Normal, benign calcification, benign mass, malignant calcification, malignant mass (5 classes)	DDSM + CBIS-DDSM (2339 images)	-	-	91	0.98

^1^ FPPI: false positive per image.

## Data Availability

The data presented in this paper are not publicly available at this time but may be obtained from the corresponding author upon reasonable request.
